# Femtosecond X-ray absorption study of electron localization in photoexcited anatase TiO_2_

**DOI:** 10.1038/srep14834

**Published:** 2015-10-06

**Authors:** F. G. Santomauro, A. Lübcke, J. Rittmann, E. Baldini, A. Ferrer, M. Silatani, P. Zimmermann, S. Grübel, J. A. Johnson, S. O. Mariager, P. Beaud, D. Grolimund, C. Borca, G. Ingold, S.L. Johnson, M. Chergui

**Affiliations:** 1 Laboratoire de Spectroscopie Ultrarapide and Lausanne Centre for Ultrafast Science (LACUS), ISIC-FSB, Ecole Polytechnique Fédérale de Lausanne, CH-1015 Lausanne, Switzerland; 2 Institut für Quantenelektronik, ETH Zürich, Wolfgang-Pauli-Str. 16, CH-8093 Zürich, Switzerland; 3 SwissFEL, Paul Scherrer Institut, CH-5232 Villigen, Switzerland; 4 Swiss Light Source, Paul Scherrer Institut, CH-5232 Villigen, Switzerland

## Abstract

Transition metal oxides are among the most promising solar materials, whose properties rely on the generation, transport and trapping of charge carriers (electrons and holes). Identifying the latter’s dynamics at room temperature requires tools that combine elemental and structural sensitivity, with the atomic scale resolution of time (femtoseconds, fs). Here, we use fs Ti K-edge X-ray absorption spectroscopy (XAS) upon 3.49 eV (355 nm) excitation of aqueous colloidal anatase titanium dioxide nanoparticles to probe the trapping dynamics of photogenerated electrons. We find that their localization at Titanium atoms occurs in <300 fs, forming Ti^3+^ centres, in or near the unit cell where the electron is created. We conclude that electron localization is due to its trapping at pentacoordinated sites, mostly present in the surface shell region. The present demonstration of fs hard X-ray absorption capabilities opens the way to a detailed description of the charge carrier dynamics in transition metal oxides.

The anatase form of Titanium dioxide (TiO_2_) is among the most commonly used in solar energy conversion processes into electrical or chemical energy^1^,^2^. These applications are entirely based on the generation of charge carriers (electrons and holes) by absorption of light, their transport and eventually, their localization by the electron-phonon coupling and/or defects. Stoichiometric anatase has an optical band gap (BG) of 3.2 eV. The Ti^4+^ (3d^0^) ions are hexacoordinated in predominantly octahedral symmetry sites having a weak D_2d_ distortion, with 4 equatorial and 2 distal Oxygen neighbour atoms at slightly different Ti-O distances. Electrons in the conduction band (CB) compete between delocalized (band-like) and localized states in the form of polarons. This competition is still debated, yet it is key to understanding the transport properties of the material^3^.

There are two categories of trapped electrons in anatase TiO_2_: those due to the presence of intrinsic Oxygen vacancies (O_vac_’s), and those resulting from photoexcitation. The former results from the removal of one neutral lattice oxygen atom that leaves two excess electrons at a point defect and three pentacoordinated Ti^4+^ ions^3^. The two excess electrons can therefore reduce two of the latter species. Previous photoemission (PE) and electron energy loss spectroscopy (EELS) studies of the surface of low temperature (LT) single crystals of the rutile and anatase forms with a controllable concentration of Oxygen vacancies (O_vac_)^4^,^5^,^6^,^7^, showed that Ti^3+^ centres occur in the form of polarons. For the anatase (101) surface, PE data always show a significant gap state ~1 eV below the Fermi level (E_F_), characteristic of a small polaron^4^,^6^,^7^. However, for the (001) plane of anatase, no such signal was observed^5^,^8^ and a delocalized, “large polaron” (~40 meV below E_F_) was reported. The small polaron formation was attributed to pentacoordinated Ti^3+^ centres, due to the presence of an O_vac_^4^,^6^,^7^. This was confirmed by scanning tunnelling microscopy/spectroscopy (STM/STS) studies of anatase TiO_2_ at 6 K, showing that small polarons form near an O_vac_ and never move^6^.

As far as photoinduced electron traps are concerned, trapping has been reported by steady-state methods, such as electron paramagnetic resonance (EPR)^9^,^10^,^11^, photoluminescence^12^, and O_2_ photodesorption^13^. In particular, low temperature (LT) EPR studies on powdered anatase TiO_2_ under continuous UV irradiation, showed the appearance of two distinct traces attributed to electrons trapped at paramagnetic Ti^3+^ sites, whose geometry remained unspecified^11^. From a comparison with the signal of the holes, the authors concluded that only a limited fraction of the CB electrons (~10% in their samples) was trapped at Ti^3+^ sites. The rest of the CB electrons remain itinerant and are EPR-silent. The nature of the photoinduced electron traps was addressed in recent theoretical studies of the photoinduced small polarons in anatase TiO_2_^14^, which predicted that for the bulk (i.e. at hexacoordinated sites), an ~80% charge localization occurs at Ti^3+^ ions. For the case of the (101) surface of anatase, polaron trapping at pentacoordinated Ti centres was found to be favourable in the sub-surface region, again with an ~80% charge localization.

In summary, it appears that the two types of localized electrons, those due to O_vac_’s^4^,^6^,^7^,^8^,^15^ and the photoinduced ones^11^,^16^, can coexist in anatase TiO_2_. While the former seem to be well established as pentacoordinated centres, it is still not clear what the nature of the photoinduced traps is and how fast trapping occurs. The knowledge of the latter delivers insight about electron transport.

Experimentally, over the past 10–20 years, ultrafast visible^17^,^18^,^19^,^20^,^21^, IR^20^,^21^,^22^, and THz^23^ spectroscopies have been used to probe the charge carrier dynamics in room temperature (RT) anatase TiO_2_ upon BG excitation. These experiments are sensitive to the overall charge dynamics in the CB and in the valence band (VB) of the material and conclusions were drawn about the mobility and trapping of the electrons and holes, in particular, that both localize at the surface within 200 fs in the case of 10–15 nm nanoparticles (NPs)^22^. Localization at the surface was assumed based on the conclusions of quasi steady-state experiments^12^,^13^, whose outcome is determined by their long time scales (on the order of μsec or longer^20^), allowing for surface reactions to occur at some point in time. However, surface trapping as such was never directly observed, let alone time-resolved. Elucidating the issues of where and how fast photoinduced electrons are trapped in TiO_2_ requires element- and geometry-sensitive time-resolved tools.

We recently implemented X-ray absorption spectroscopy (XAS) with 80 ps time resolution at the Ti K-edge of RT colloidal solutions of TiO_2_ NPs. XAS is element-specific and it also provides information about the local geometry around specific atoms, and about their electronic structure, as it probes the valence orbitals. Our results on ~20 nm bare anatase and amorphous NPs showed that the photogenerated electrons form Ti^3+^ centres, with nearly a full electron charge localizing on them^16^, as inferred from the edge shift of ~−1 eV and in good agreement with the theoretical predictions^14^. The Ti^3+^ centres decayed in a bi-exponential mode over 100 s of ps to a few ns. Because anatase TiO_2_ NPs are known to have an ordered core with a defect-rich surface shell containing a high proportion of under-coordinated Ti centres^24^ and since the spectral changes pointed to the reduced centres being in an amorphous-like environment, it was also concluded that these traps are mostly localized in the shell region, and we tentatively assigned them to distorted hexa-coordinated and to penta-coordinated sites, based on the intensity enhancement of the dipole-forbidden 1 s-3d pre-edge transitions induced by symmetry breaking. The 80 ps time resolution of the experiment was not sufficient to determine the migration and trapping times of the electron. This is important in order to elucidate how the Ti^3+^ centres are created. In particular, given the coexistence of itinerant and localized electrons^4^,^6^,^25^, one may ask if localization is preceded by migration and for how long? In addition, in the event of small polaron formation^14^, the electron-phonon interaction leads to lattice relaxation which is confined to a single structural unit. Whether this polaron formation has an extrinsic (formation assisted by defects) or intrinsic (simply due to electron-phonon coupling) nature^25^ is still unclear.

Here, we present the first femtosecond (fs) Ti K-edge X-ray absorption near-edge spectroscopy (XANES) measurements of electron localization in photoexcited RT anatase TiO_2_ colloidal NPs (see Fig. S1 for their X-ray powder diffraction pattern). We used the fs-slicing scheme at the Swiss Light Source, achieving a temporal resolution of ~200 fs^26^. Excitation of the electron to the CB is carried out by indirect band gap absorption^27^ using 355 nm (3.49 eV), 150 fs pump pulses. Details of the experiment and sample handling are given in the Supplementary Material section.

Figure 1a shows the steady-state Ti K-edge spectrum of TiO_2_, already discussed in refs 16,24, with the edge at ~4.985 keV and the weakly allowed pre-edge features, labelled A_1_-A_3_ and B, around 4.97 keV. Figure 1b reproduces from ref. 16, the transient spectrum (X-ray absorbance difference between excited and unexcited sample) recorded 100 ps after excitation at 355 nm (3.49 eV). The dipole-forbidden 1s-3d transitions are weakly allowed in anatase because of partial 3d-4p orbital (dipole-quadrupole) mixing, caused by the lowering of symmetry in the D_2d_ trapping site of hexacoordinated Ti^4+^ ions. Additional symmetry lowering enhances them furthermore. As a matter of fact, this is the case in the 100 ps transient, while the ≤1eV red shift of the edge is manifested by the strong absorption increase at 4.982 keV. The transient recorded at 1 ps time delay is also shown in Fig. 1b. It is similar to the 100 ps transient taking into account the different excitation yields (§ S.3) but the data are much noisier due to the 4 orders of magnitude lower X-ray flux in the slicing scheme compared to the ps experiments.

Figure 2 shows the temporal profile of the signal at maximum (4.982 keV), which maps the evolution of the population of reduced Ti sites. It shows a prompt rise within 200 fs, reaching a level that remains constant up to the limit of our scan (50 ps, Fig. S2). The data were fitted to a model (§ S.4), which takes into account a single rate, convoluted with a Gaussian of 200 fs width to account for the time resolution of the experiment. The best fit delivers a rise time of 170 fs, after deconvolution. However, given the noise and large error bars, we could still fit the data with a maximum rise time of 300 fs (Fig. 2). The similarity of the transient spectra (Fig. 1b) and the fact that the signal shows no evolution beyond 0.5 ps, suggest that the traps observed at <1 ps are the same as those previously reported at 100 ps^16^, implying a prompt localization of the electrons with little migration.

Indeed, an estimate of the diffusion length 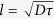, of the electrons can be obtained from the diffusion coefficient *D* ≈ 10^−6^ m^2^/s of anatase TiO_2_^28^. For a rise time of 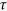
=170 to 300 fs, we find that *l*~4.0–5.5 Å. This result implies that the electron is localized within or very near the unit cell in which it was photogenerated. In addition, given the −1 eV shift of the edge, a near full electron charge localization occurs. Of course, there is a large uncertainty on the value of D, but even an increase by one order of magnitude would only increase the distance by a factor of three, which would not change the above conclusions. An increase by two orders of magnitude, which is unrealistic, would also not alter the conclusions significantly, i.e. that the electron does not migrate a long distance and least of all, distances of the order of the NP size. On the other hand, assuming the above value of D, travelling from the centre of the NP to the surface of the 20 nm diameter particles would require ~100 ps.

The size of the NPs is much smaller than the penetration depth of the 355 nm beam in TiO_2_^29^, so that their entire volume is excited. While defects may also occur in the core region of the NPs, most are known to be concentrated in the surface shell region^24^,^30^. We therefore conclude that the photogenerated Ti^3+^ centres are largely located in the shell region.

As already mentioned, the pre-edge 1s-3d transitions (Fig. 1a) are very sensitive to symmetry changes of the Ti sites, and in particular, the A_2,3_ features^31^. The distortion calculated by Di Valentin *et al.*^3^,^14^ for bulk (i.e. hexacoordinated) polarons does not break the local symmetry and it therefore cannot lead to enhanced pre-edge A_2,3_ bands. Furthermore, an asymmetric distortion where Ti-O bond lengths increase around a hexacoordinated Ti^3+^ centre^3^,^14^, will also not give rise to an increased intensity of the pre-edge features as was demonstrated in ref. 32.

In particular, it is useful to cast the present results in the context of past studies. Three observations from our previous ps XAS study^16^ need to be recalled here: i) the transients reflect a shift of spectral weight from anatase-like to reduced amorphous-like features; ii) the biexponential decay kinetics of the signal in photoexcited anatase suggests the presence of two species that we therefore distinguish kinetically but not spectroscopically; iii) the decay times and pre-exponential factors are quite similar in anatase and amorphous NPs. Furthermore, several experimental studies and simulations on amorphous TiO_2_ NPs^33^,^34^,^35^ conclude that the average coordination number of Ti atoms is between 5 and 6, while anatase NPs have a shell region with a greater proportion of 5-fold coordination^24^. Thus, the most likely trapping sites are the pentacoordinated ones, which are dominant in the shell region. As mentioned above pentacoordination resulting from an O_vac_ leaves two excess electrons and three undercoordinated Ti atoms. If two of the Ti atoms are reduced by the two excess electrons, the most likely recipient of the photogenerated electron is the third undercoordinated Ti^4+^ centre.

This ties in nicely with the following observations^16^: a) pentacoordination leads to an enhancement of the pre-edge features even if the Ti-O bond lengths increase for polaron formation^32^; b) The presence of two inequivalent O neighbours in the D_2d_ sites of anatase TiO_2_, implies two possible pentacoordinated sites. As already mentioned, two Ti^3+^ traps had been reported in LT EPR studies^11^, and the biexponential decay kinetics of the transient ps XAS signals also points to two Ti^3+^ defects^16^; c) in the latter, the ratio of pre-exponential factors is ~2.4, very close to the expected ratio of 2, if we assume a statistical distribution of reduced pentacoordinated sites. Therefore, we conclude that the most likely trapping sites of the photoinduced electron are pentacoordinated Ti centres, which are typically of two types, as they results from either the vacancy of either an equatorial or a distal Oxygen.

Turning now to the mechanism of electron localization, the physics of charge localization in materials is a complex problem^25^, and more so in the case of TiO_2_, as it arises from the interplay between different localizing effects, such as the electron-phonon interaction or weak potentials generated by static lattice imperfections^36^. Even in this case, the question arises whether a transition from a large to a small polaron occurs, or if a small polaron is formed which then migrates to become trapped at a defect. From the above, we exclude the latter case, due to the prompt trapping reported here.

In the presence of intrinsic or extrinsic defects, polaron self-trapping and trapping by the weak potential of static lattice imperfections combine to freeze the motion of the carriers^25^,^36^, even when a single of these mechanisms is in itself not sufficient to induce charge localization. The present scenario in which the electron is trapped in or near the same unit cell where it was created implies that the pre-existing defects (mostly in the shell region) are dominating the trapping dynamics, despite the intermediate-to-strong electron-phonon coupling of anatase TiO_2_^37^. This is even more so that under our present fluences (see Supplementary Material)^16^,^29^, a Fermi liquid probably forms in the CB. The scenario in which electron-phonon coupling is operative would also be an ultrafast process given the period of the E_u_ antiphase breathing mode of the oxygen atoms (oscillation period of ~40 fs)^5^,^38^,^39^. Thus, whichever mechanism is in play (direct trapping or mediated by electron-phonon coupling), electron trapping at defects is ultrafast.

Finally, the present results allow us to make predictions on the dynamics of charge injection and trapping in the case of dye-sensitised solar cells. Since the injected electron is trapped at the outer surface (the most defective part of the device)^16^,^24^ and since trapping is an ultrafast process, this means that the electron is most likely trapped in the vicinity of the cationic dye it stems from. This does not mean that it will remain there after the initial trapping. In addition, although not all electrons are trapped at the surface, or else the DSSCs would not function, this represents a limitation to the functioning devices for conversion of solar light into electricity. However, it represents an advantage for photocatalysis, depending on certain conditions determined by the trap energy.

The present first demonstration of femtosecond X-ray absorption spectroscopy applied to room temperature metal oxide nanoparticles opens the way to their full characterization with higher time resolution and flux, which is now achievable at free electron lasers^40^.

## Additional Information

**How to cite this article**: Santomauro, F. G. *et al.* Femtosecond X-ray absorption study of electron localization in photoexcited anatase TiO2. *Sci. Rep.* **5**, 14834; doi: 10.1038/srep14834 (2015).

## Supplementary Material

Supplementary Information

## Figures and Tables

**Figure 1:**
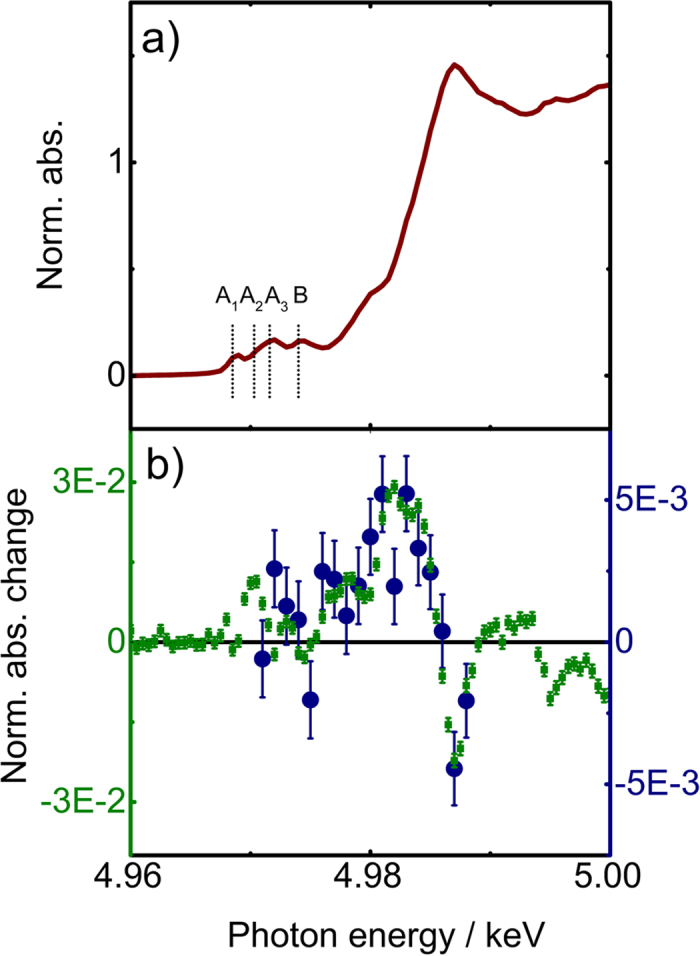
(**a**) Normalized static Ti K-edge XANES spectrum of colloidal nanoparticles of anatase TiO_2_ at room temperature. (**b**) Transient (difference) Ti K-edge XANES spectra (difference of the excited minus the unexcited sample absorption) for 355 nm excitation of colloidal nanoparticles of anatase TiO_2_, recorded at time delays of 100 ps (green squares, left vertical axis)^16^ and 1 ps (this work, blue dots, right vertical axis).

**Figure 2:**
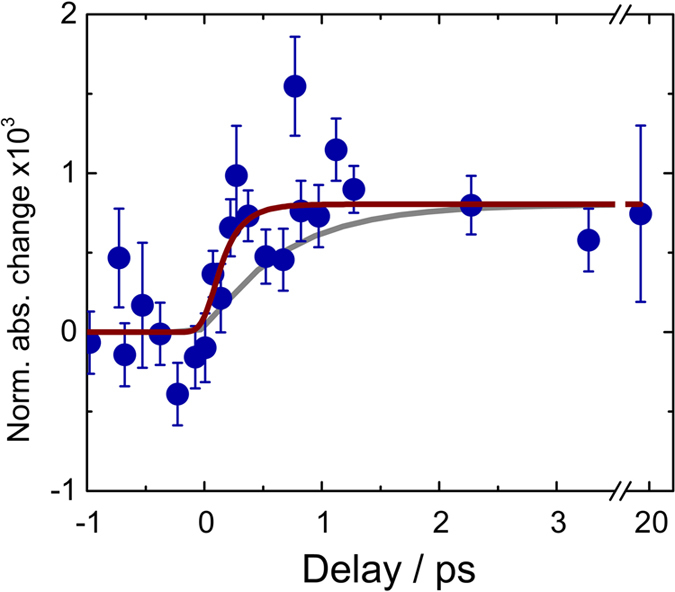
Temporal evolution of the photo-induced X-ray absorption change at 4.982 keV, of room temperature colloidal TiO_2_ nanoparticles excited at 355 nm (blue dots). After the rise, the signal remains constant up to the limit of our time scan (50 ps, see Fig. S2). The brown trace represents the best fit of the data, yielding a rise time of 170 fs (see § S4). The grey trace shows a satisfactory fit of the data with the longest rise time of 300 fs that represents an upper limit.

## References

[b1] Nakata K. & Fujishima A. TiO2 photocatalysis: Design and applications. J Photoch Photobio C 13, 169–189, 10.1016/j.jphotochemrev.2012.06.001 (2012).

[b2] Kalyanasundaram K. & Gratzel M. Applications of functionalized transition metal complexes in photonic and optoelectronic devices. Coordin Chem Rev 177, 347–414 (1998).

[b3] Di Valentin C., Pacchioni G. & Selloni A. Reduced and n-Type Doped TiO2: Nature of Ti3+ Species. J Phys Chem C 113, 20543–20552, 10.1021/Jp9061797 (2009).

[b4] Egdell R. G., Eriksen S. & Flavell W. R. Oxygen Deficient Sno2(110) and Tio2(110) - a Comparative-Study by Photoemission. Solid State Commun 60, 835–838, 10.1016/0038-1098(86)90607-1 (1986).

[b5] Moser S. *et al.* Tunable Polaronic Conduction in Anatase TiO2. Phys Rev Lett 110, Artn 196403, 10.1103/Physrevlett.110.196403 (2013). 23705725

[b6] Setvin M. *et al.* Direct View at Excess Electrons in TiO2 Rutile and Anatase. Phys Rev Lett 113, Artn 086402, 10.1103/Physrevlett.113.086402 (2014). 25192111

[b7] Eriksen S. & Egdell R. G. Electronic Excitations at Oxygen Deficient Tio2(110) Surfaces - a Study by Eels. Surf Sci 180, 263–278, 10.1016/0039-6028(87)90048-3 (1987).

[b8] Thomas A. G. *et al.* Comparison of the electronic structure of anatase and rutile TiO2 single-crystal surfaces using resonant photoemission and x-ray absorption spectroscopy. Phys Rev B 75, Artn 035105, 10.1103/Physrevb.75.035105 (2007).

[b9] Szczepankiewicz S. H., Moss J. A. & Hoffmann M. R. Slow surface charge trapping kinetics on irradiated TiO2. J Phys Chem B 106, 2922–2927, 10.1021/Jp004244h (2002).

[b10] Dimitrijevic N. M., Saponjic Z. V., Rabatic B. M., Poluektov O. G. & Rajh T. Effect of size and shape of nanocrystalline TiO2 on photogenerated charges. An EPR study. J Phys Chem C 111, 14597–14601, 10.1021/Jp0756395 (2007).

[b11] Berger T. *et al.* Light-induced charge separation in anatase TiO2 particles. J Phys Chem B 109, 6061–6068, 10.1021/Jp0404293 (2005). 16851666

[b12] Mercado C. C. *et al.* Location of Hole and Electron Traps on Nanocrystalline Anatase TiO2. J Phys Chem C 116, 10796–10804, 10.1021/Jp301680d (2012).

[b13] Thompson T. L. & Yates J. T. Monitoring hole trapping in photoexcited TiO2(110) using a surface photoreaction. J Phys Chem B 109, 18230–18236, 10.1021/Jp0530451 (2005). 16853345

[b14] Di Valentin C. & Selloni A. Bulk and Surface Polarons in Photoexcited Anatase TiO2. J Phys Chem Lett 2, 2223–2228, 10.1021/Jz2009874 (2011).

[b15] Sekiya T. *et al.* Defects in anatase TiO2 single crystal controlled by heat treatments. J Phys Soc Jpn 73, 703–710, 10.1143/Jpsj.73.703 (2004).

[b16] Rittmann-Frank M. H. *et al.* Mapping of the Photoinduced Electron Traps in TiO2 by Picosecond X-ray Absorption Spectroscopy. Angew Chem Int Edit 53, 5858–5862, 10.1002/anie.201310522 (2014). 24820181

[b17] Skinner D. E., Colombo D. P., Cavaleri J. J. & Bowman R. M. Femtosecond Investigation of Electron Trapping in Semiconductor Nanoclusters. J Phys Chem-Us 99, 7853–7856, 10.1021/J100020a003 (1995).

[b18] Colombo D. P. & Bowman R. M. Femtosecond Diffuse-Reflectance Spectroscopy of Tio2 Powders. J Phys Chem-Us 99, 11752–11756, 10.1021/J100030a020 (1995).

[b19] Serpone N., Lawless, D. & Khairutdinov, R. Size Effects on the Photophysical Properties of Colloidal Anatase Tio2 Particles - Size Quantization or Direct Transitions in This Indirect Semiconductor. Journal of Physical Chemistry 99, 16646–16654, 10.1021/J100045a026 (1995).

[b20] Yoshihara T. *et al.* Identification of reactive species in photoexcited nanocrystalline TiO2 films by wide-wavelength-range (400-2500 nm) transient absorption spectroscopy. J Phys Chem B 108, 3817–3823, 10.1021/Jp031305d (2004).

[b21] Furube A. *et al.* Femtosecond visible-to-IR spectroscopy of TiO2 nanocrystalline films: Dynamics of UV-generated charge carrier relaxation at different excitation wavelengths - art. no. 66430J. Physical Chemistry of Interfaces and Nanomaterials Vi 6643, J6430–J6430, 10.1117/12.733316 (2007).

[b22] Tamaki Y. *et al.* Dynamics of efficient electron-hole separation in TiO2 nanoparticles revealed by femtosecond transient absorption spectroscopy under the weak-excitation condition. Phys Chem Chem Phys 9, 1453–1460, 10.1039/B617552j (2007). 17356752

[b23] Nemec H., Kuzel P. & Sundstrom V. Charge transport in nanostructured materials for solar energy conversion studied by time-resolved terahertz spectroscopy. J Photoch Photobio A 215, 123–139, 10.1016/j.jphotochem.2010.08.006 (2010).

[b24] Luca V. Comparison of Size-Dependent Structural and Electronic Properties of Anatase and Rutile Nanoparticles. J Phys Chem C 113, 6367–6380, 10.1021/Jp808358v (2009).

[b25] Suzuki S. & Toyozawa Y. Coexistence of Itinerant Electrons and Self-Trapped Electrons. J Phys Soc Jpn 59, 2841–2847, 10.1143/Jpsj.59.2841 (1990).

[b26] Beaud P. *et al.* Spatiotemporal stability of a femtosecond hard-X-ray undulator source studied by control of coherent optical phonons. Phys Rev Lett 99, Artn 174801, 10.1103/Physrevlett.99.174801 (2007). 17995338

[b27] Emori M., Sugita M., Ozawa K. & Sakama H. Electronic structure of epitaxial anatase TiO2 films: Angle-resolved photoelectron spectroscopy study. Phys Rev B 85, Artn 035129, 10.1103/Physrevb.85.035129 (2012).

[b28] Enright B. & Fitzmaurice D. Spectroscopic determination of electron and mole effective masses in a nanocrystalline semiconductor film. J Phys Chem-Us 100, 1027–1035, 10.1021/Jp951142w (1996).

[b29] Matsuzaki H. *et al.* Photocarrier dynamics in anatase TiO2 investigated by pump-probe absorption spectroscopy. J Appl Phys 115, Artn 053514, 10.1063/1.4864219 (2014).

[b30] Hanley T. L., Luca V., Pickering I. & Howe R. F. Structure of titania sol-gel films: A study by X-ray absorption spectroscopy. J Phys Chem B 106, 1153–1160, 10.1021/Jp012225h (2002).

[b31] Farges F., Brown G. E. & Rehr J. J. Ti K-edge XANES studies of Ti coordination and disorder in oxide compounds: Comparison between theory and experiment. Phys Rev B 56, 1809–1819, 10.1103/PhysRevB.56.1809 (1997).

[b32] Jiang N., Su D. & Spence J. C. H. Determination of ti coordination from pre-edge peaks in TiK-edge XANES. Phys Rev B 76, Artn 214117, 10.1103/Physrevb.76.214117 (2007).

[b33] Petkov V., Holzhuter G., Troge U., Gerber T. & Himmel B. Atomic-scale structure of amorphous TiO2 by electron, x-ray diffraction and reverse Monte Carlo simulations. J Non-Cryst Solids 231, 17–30, 10.1016/S0022-3093(98)00418-9 (1998).

[b34] Prasai B., Cai B., Underwood M. K., Lewis J. P. & Drabold D. A. Properties of amorphous and crystalline titanium dioxide from first principles. J Mater Sci 47, 7515–7521, 10.1007/s10853-012-6439-6 (2012).

[b35] Zhang H. Z., Chen B., Banfield J. F. & Waychunas G. A. Atomic structure of nanometer-sized amorphous TiO2. Phys Rev B 78, 214106, Artn 214106, 10.1103/Physrevb.78.214106 (2008).

[b36] Stoneham A. M. *et al.* Trapping, self-trapping and the polaron family. J Phys-Condens Mat 19, Artn 255208, 10.1088/0953-8984/19/25/255208 (2007).

[b37] Jacimovic J. *et al.* Pressure dependence of the large-polaron transport in anatase TiO2 single crystals. Epl-Europhys Lett 99, Artn 57005, 10.1209/0295-5075/99/57005 (2012).

[b38] Gonzalez R. J. & Zallen R. Optical studies of nanophase titania. Nato Asi 3 High Tech 23, 395–403 (1997).

[b39] Deskins N. A. & Dupuis M. Electron transport via polaron hopping in bulk TiO2: A density functional theory characterization. Phys Rev B 75, Artn 195212, 10.1103/Physrevb.75.195212 (2007).

[b40] Bencivenga F. *et al.* Nanoscale dynamics by short-wavelength four wave mixing experiments. New J Phys 15, Artn 123023, 10.1088/1367-2630/15/12/123023 (2013).

