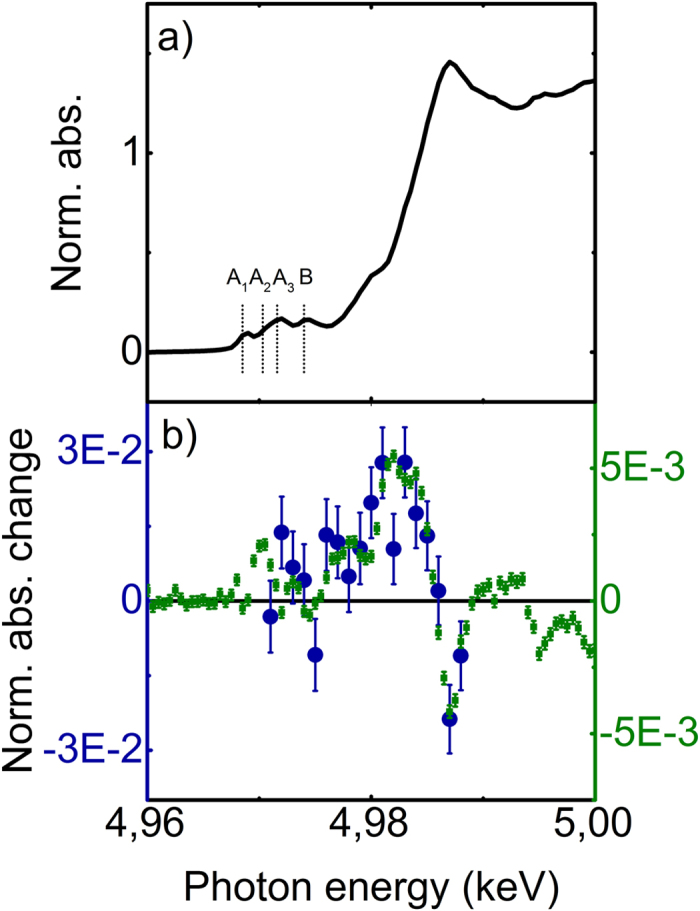# Corrigendum: Femtosecond X-ray absorption study of electron localization in photoexcited anatase TiO_2_

**DOI:** 10.1038/srep27228

**Published:** 2016-06-10

**Authors:** F. G. Santomauro, A. Lübcke, J. Rittmann, E. Baldini, A. Ferrer, M. Silatani, P. Zimmermann, S. Grübel, J. A. Johnson, S. O. Mariager, P. Beaud, D. Grolimund, C. Borca, G. Ingold, S. L. Johnson, M. Chergui

Scientific Reports 5: Article number: 1483410.1038/srep14834; published online: 10062015; updated: 06102016.

This Article contains errors in Figure 1: the coloured axes have been inverted. The correct Figure 1 appears below. As a result, the Figure legend,

“recorded at time delays of 100 ps (green squares, left vertical axis)^16^ and 1 ps (this work, blue dots, right vertical axis).”

should read:

“recorded at time delays of 100 ps (green squares, right vertical axis)^16^ and 1 ps (this work, blue dots, left vertical axis).”[Fig f1]

## Figures and Tables

**Figure 1 f1:**