# Supplementary material for: Femtosecond X-ray absorption study of electron localization in photoexcited anatase TiO2
Source: Sci Rep. 2015 Oct 6;5:14834. doi: 10.1038/srep14834 (PMC4594303; doi:10.1038/srep14834)
Supplement: Supplementary Information [file srep14834-s1.pdf]

Supplemental Material
Real-time probing of electron localization in photoexcited anatase TiO_2

F. G. Santomauro[1], A. Lübcke[1 §], J. Rittmann[1], E. Baldini[1], A. Ferrer[2,4], M. Silatani[1], P. Zimmermann[1 ç], S. Grübel[2,4], J. A. Johnson[4], S. O. Mariager[3], P. Beaud[3,4], D. Grolimund[4], C. Borca[4], G. Ingold[3,4], S.L. Johnson[2] and M. Chergui[1]

[1]Laboratoire de Spectroscopie Ultrarapide, ISIC-FSB, Ecole Polytechnique Fédérale de Lausanne, CH-1015 Lausanne, Switzerland

E-mail: majed.chergui@epfl.ch

[2]Institut für Quantenelektronik, ETH Zürich, Wolfgang-Pauli-Str. 16, CH-8093 Zürich, Switzerland

[3]SwissFEL, Paul Scherrer Institut, CH-5232 Villigen, Switzerland

[4]Swiss Light Source, Paul Scherrer Institut, CH-5232 Villigen, Switzerland

§ Now at Max-Born-Institut for nonlinear optics and short pulse spectroscopy, Max-Born Strasse 2A, 12489 Berlin, Germany

ç Now at Department of Inorganic Chemistry and Catalysis, Utrecht University, Universiteitsweg 99, 3584 CG Utrecht, Netherlands

S.1 Experimental procedures

The experiments are performed at the X05LA (microXAS) beamline at the Swiss Light Source, Paul Scherrer Institute, using the fs-slicing scheme.[1] The slicing source is operated at 2 kHz and the fs X-ray slice is spatially separated from the core beam by slits. It passes through the first mirror (M1) and is vertically collimated onto a Ge(111) double crystal monochromator. The energy range in the experiment is from 4.97 keV to 4.99 keV, at an energy resolution of <2 eV. The X-ray pulses are focused by a Kirkpatrick-Baez system to a spot size of 30 x 30 μm^2 (FWHM). At the sample position, a single X-ray slice in this energy range contains ~12 photons per pulse, resulting in a flux five orders of magnitude lower than in our previous XAS experiments on TiO_2 with 80 ps time resolution.[2] The entire data in figure 2 has been accumulated over ~10000 s per data point. Excitation pulses are supplied by a regeneratively pumped Ti:sapphire laser system at 1 kHz repetition rate and pulse energies of about 1.7 mJ (1.2 mJ after the transfer line to the X-ray hutch) at 800 nm (1.55 eV). The difference in repetition rate between optical

pump and X-ray probe pulses combined with a single shot data acquisition allows to measure the photo-induced differences of the electronic structure on a pulse-to-pulse basis. Pulses are split at variable pulse energies employing a half wave plate and a thin film polarizer. One part is used to pump an optical parametric amplifier (TOPAS) to provide pulses at 355 nm (after two frequency doubling stages). These pulses are focused by a spherical UV-enhanced aluminum mirror into a 60x80 μm^2 spot on the sample at a power of 16 mW. The remaining part of the 800 nm light is transferred via a delay line and focused by the same spherical mirror onto the same spot as the 355 nm pulses. Difference frequency generation in a nonlinear β-Barium Borate (BBO) crystal is used to find the temporal overlap between the UV and the 800 nm pulses. The 800 nm pulses were required to find the temporal overlap with the X-ray probe pulse. This is achieved in consecutive steps: 1) A fast photodiode measures the rough timing between the X-ray core beam and the 800 nm laser pulse with a precision of few ps; 2) The temporal overlap between the X-ray and 800 nm pulses is found by recording the coherent optical phonons in a superlattice of $(Ba_{0.7}Sr_{0.3})TiO_3$ and $(La_{0.7}Sr_{0.3})MnO_3$;[3] 3) The difference frequency generation in a BBO crystal ensures temporal overlap between the 800 nm and 355 nm pulses. The X-ray pulse duration is about 140 fs, *i.e.* the temporal resolution of the experiment is on the order of 200 fs. X-ray absorption is measured in transmission mode with an avalanche photodiode. The pump pulses have a pulse duration of ~150 fs. They are focused to spot sizes of (60 x 80 μm^2 FWHM) onto a 100 µm thick, flat liquid jet of sample solution. The nanoparticle concentration is adjusted for an OD of 1.5 at 355 nm. The OD of the suspension is affected by scattering effects from the TiO_2 NPs, which means that the effective absorption coefficient of the system is lower. We used a fluence of ~340 mJ/cm^2, which is higher than in our previous work and, correspondingly, results in a significantly higher excitation yield. The flow speed of the liquid jet, which is 4 m/s, allows for continuous renewal of the sample between pump/probe pulse pairs. All the x-ray measurements were run at room temperature and ambient pressure.

S.2 Sample synthesis and characterization

The sample is a colloidal suspension of 20 nm large TiO_2 nanoparticles in acetic water. The suspension is prepared via a sol-gel synthesis, which is carried out in an inert atmosphere using a glove box. The precursor used for the NPs preparation is Titanium isopropoxide (Sigma Aldrich, 99.999% purity). The solution obtained mixing the precursor in 250 ml H_2O 18 MΩ and 80 ml of acetic acid was peptized at 80 °C for about 2 h until turned into a transparent gel. The sample was then heated at 230 °C in an autoclave for 12 hours. The final solution was characterized via UV-Vis spectroscopy to adjust the concentration and XRD (Bruker D8 Advance diffractometer) in order to establish the crystal structure (Fig. S1). From the reproducibility of the synthesis we expect an average diameter for anatase TiO_2 of about 20 nm as in our previous work. For more details about the sample preparation and characterization using UV-Vis spectroscopy, dynamics light scattering and TEM see the supporting information of ref. [2].

S.3 Estimate of the excitation yield

The excitation yield *f* has been estimated using the same approach as in ref. [2]. The sample concentration is c = 833 mM and the thickness of the liquid jet d = 0.010 cm. Using the following equation:

$$f = \frac{N_{ph.}}{N_A \cdot c \cdot \mathrm{vol.}} \left(1 - 10^{(-\epsilon \cdot c \cdot d)}\right)$$

Where N_{ph} gives the number of laser photons, vol. describes the volume irradiated by the laser and ε is the extinction coefficient at the excitation wavelength (355 nm). For the experimental conditions used, where N_{ph}=3 10^{13}, ε_{355nm}=180 $M^{-1}cm^{-1}$ and vol.=4.8 10^{-7} cm^3,

we find an excitation yield f~12% which is about 6 times higher than in our previous work.[2]

S.4 Fit function

The measured time traces are analyzed by fitting a rate model to the data, taking into account a single growth rate k. To account for the limited time resolution, the solution of the rate equation is convoluted with a Gaussian function of width W= 200 fs, describing the cross-correlation of optical pump and X-ray probe pulses. The time-dependent signal can thus be described by:

$$f(t) = \frac{1}{2} A \cdot \left\{ \text{Erfc}\left[-\sqrt{2} \cdot \frac{t - t_0}{W} \right] - e^{\frac{1}{8}k^2 w^2} \cdot e^{-k(t-t_0)} \cdot \text{Erfc}\left[\frac{kW}{2\sqrt{2}} - \sqrt{2} \cdot \frac{t - t_0}{W} \right] \right\}$$

where A is the maximum transient signal and t_0 is the time at which pump and probe pulses overlap.

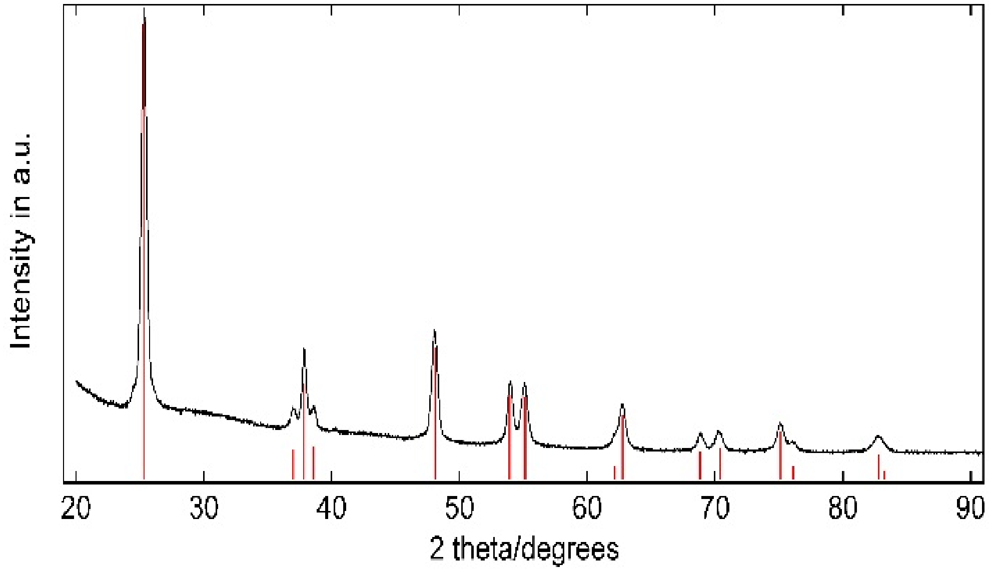

FIG. S1: XRD pattern of the dried sample (black trace). The red sticks represent the diffraction reference data of anatase TiO_2 at 25 °C.[4]

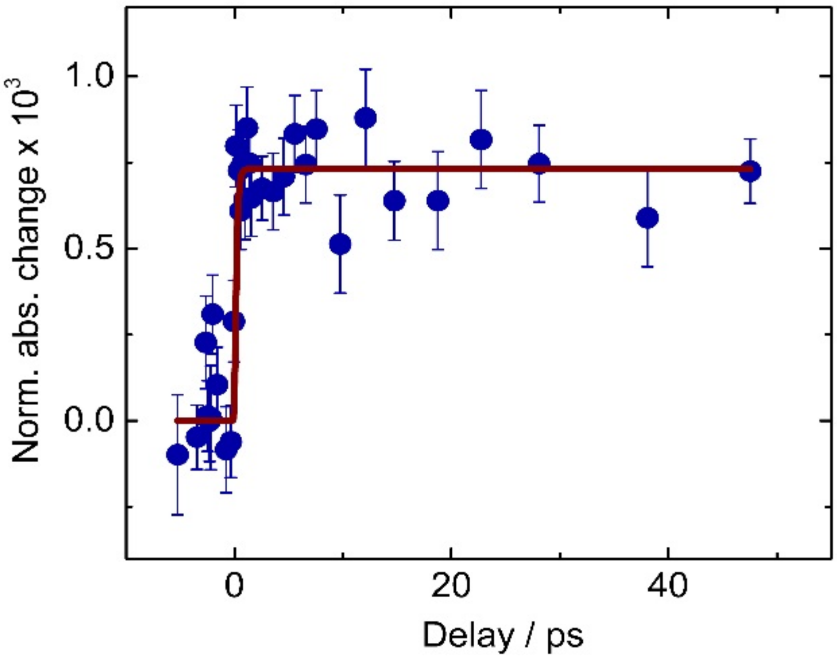

FIG. S2: Temporal evolution of the photoinduced X-ray absorbance change at 4.982 keV, monitoring the localization of electrons at Ti^{4+} sites thus forming Ti^{3+} ones. The signal stays constant for about 50 ps.

---

[1] P. Beaud *et al.*, Phys Rev Lett **99** (2007).

[2] M. H. Rittmann-Frank, C. J. Milne, J. Rittmann, M. Reinhard, T. J. Penfold, and M. Chergui, Angew Chem Int Edit **53**, 5858 (2014).

[3] C. V. K. Schmising, M. Bargheer, M. Kiel, N. Zhavoronkov, M. Woerner, T. Elsaesser, I. Vrejoiu, D. Hesse, and M. Alexe, Appl Phys B-Lasers O **88**, 1 (2007).

[4] C. J. Howard, T. M. Sabine, and F. Dickson, Acta Crystallogr B **47**, 462 (1991).
